# Training Program in Population Neuroscience of Alzheimer’s Disease and Age-Related Dementias

**DOI:** 10.1093/geroni/igaa057.009

**Published:** 2020-12-16

**Authors:** Caterina Rosano

**Affiliations:** University of Pittsburgh, Pittsburgh, Pennsylvania, United States

## Abstract

The recent successes of medical science in extending lifespan, with marginal improvements in healthspan, have increased the number of adults reaching very old ages, but also their burden of age-related comorbidities. For these “new” populations of older adults, cumulative exposure to chronic conditions, biological and chronological aging as well as life-long environmental factors, interact with each other in ways that are both very complex and not yet well understood. Understanding these complex pathways and their contribution to brain aging is fundamentally important to conduct rigorous etiological research into the causes of ADRD. We are also seeing great technological advances in measuring health factors in general and brain characteristics in particular, the application of which is providing ever more precise phenotypes but also very large and complex datasets. Such “big” data require careful sampling designs and analytical approaches infused with an understanding of the condition being studied to effectively produce new knowledge to move research to treatment and prevention. We propose that the successful clinical neuroepidemiological investigators of the future must be able to link comorbidities, environmental exposures, lifestyles, genomics, e.g. host susceptibility, with knowledge of modern technology of neurosciences and measurement of brain disease and data science. We will describe our experience at the University of Pittsburgh in leading a new training program in Population Neuroscience of ADRD. Our curriculum responds to the changing landscape of career pathways, technological innovations, and demographic shifts in the aging population.

